# Are school settings restricting access to daily physical activity for children with cystic fibrosis? Parents’ perspectives and recommendations for practice

**DOI:** 10.1080/17482631.2024.2419165

**Published:** 2024-10-22

**Authors:** Emma Powell, Lorayne A Woodfield, Alexander J Powell, Tony D Myers, Miranda Barker

**Affiliations:** a Faculty of Education, Birmingham Newman University, Birmingham, UK; b Faculty of Arts, Society and Professional Studies, Birmingham Newman University, Birmingham, UK

**Keywords:** Physical activity, cystic fibrosis, school, parents, experiences, inclusion

## Abstract

**Background:**

Cystic Fibrosis (CF) is a genetic life limiting disease that impacts upon quality of life. An aim of CF care is to preserve lung function, with physical activity (PA) being an important part of daily airway clearance. Ensuring children have opportunities to engage in PA at school should be an important part of their daily routine. It is important to gain parental perspectives on this, as they manage the daily care for their children. This study aims to explore parents’ perceptions of school-based PA for their children with CF.

**Methods:**

Parents of children with CF (*n* = 10), from three regions of the UK (England, Wales and Northern Ireland) took part in online semi-structured interviews. Data were analysed using Interpretative Phenomenological Analysis (IPA).

**Results:**

Although parents recognized the benefits of school-based PA for their children, systemic barriers in the school setting often inhibit daily PA for children with CF, including teachers’ misconceptions, emotional and physical barriers, and PA not being a priority.

**Conclusion:**

Recommendations for practice have been developed to help engage children with CF in daily school-based PA in an inclusive way, with the hope of maintaining health outcomes for children with CF.

## Introduction

Cystic Fibrosis (CF) is an inherited, life-limiting disease caused by a fault in the Cystic Fibrosis Transmembrane Conductor (CFTR) gene that affects the movement of salt and water in and out of the body’s cells (Roda et al., 2022). This dysfunction results in a build-up of thick sticky mucus which disrupts airway clearance leading to bacterial colonization and lung damage (Roda et al., 2022). CF can also affect other organs in the body including, but not limited to, the pancreas and liver. There are currently over 90,000 people worldwide (Roda et al., 2022) and 10,908 people in the UK living with CF (Cystic Fibrosis Trust, 2023), and despite there being advances in treatment such as modulator therapies, the disease continues to limit survival, impacts upon quality of life and results in a huge burden of daily care for people with CF and their families (Bell et al., 2020). The median predicted survival age for people born today with CF in the UK is 53.3 years. This means that half of this population is predicted to die before they reach this age. The majority (61.7%) of CF deaths in the UK in 2021 were respiratory related (Cystic Fibrosis Trust, 2023). Therefore, one of the main aims of CF care is to preserve lung function and to utilize multidisciplinary teams to assist with this including consultants, nurses, physiotherapists, dietitians and psychologists (Cystic Fibrosis Trust, 2023).

Daily physiotherapy for children with CF helps to clear the thick mucus from their lungs. A range of techniques are often used including active breathing cycles, pep devices and physical activity (PA) (Cystic Fibrosis Trust, 2023). A systematic review (Puppo et al., 2020) of PA in children with CF highlights PA as an important part of care, as engaging in high levels of PA is associated with improved airway clearance, an increase in lung function and improvements in bone density, along with high engagement levels of PA being positively associated with better health-related quality of life. PA is defined as “any bodily movement produced by skeletal muscles” (Caspersen et al., 1985), and the World Health Organization (Bull et al., 2020) recommends that school-aged children should participate in at least 60 min of moderate-to-vigorous PA (MVPA) daily. For children with CF, these PA guidelines are applicable and important (Southern et al., 2024). The recently updated European Cystic Fibrosis Society (ECFS) standards for care of people with CF (Southern et al., 2024), reinforce the importance of physical activity to facilitate mucociliary clearance. It also highlights that a personal approach should be taken, however people with CF should aim to maximize their PA. Further, the UK government’s statutory guidance on supporting pupils at school with medical conditions states that children with medical conditions should be properly supported at school (Department for Education, 2014b). However, there is currently limited guidance for schools on the promotion of PA for children with CF, and they can often fall into the “protected child syndrome” in which they are more likely to be encouraged to be sedentary than engage in PA (Thornton, 1997); which could be further detrimental to the health of children with CF.

Considering the time children spend at school in England (32.5 h a week) (Length of the school week: non statutory guidance [Internet], Department for Education (DfE) 2023) and the daily treatment needed in the care of children with CF, the school environment provides a key opportunity for children to engage in PA as part of their daily CF care. Additionally, one of the key messages from the Lancet’s Respiratory Medicine Commission on the future of CF care outlines that we need to consider management approaches that help to maintain health whilst also minimizing the burden of care for patients and their families (Bell et al., 2020). Therefore, the school environment could create an opportunity to contribute to the daily healthcare needs of children with CF whilst also reducing some of the burden from families, especially since the UK government recommends that all children should accumulate at least 30 min of daily PA in the school setting (HM Government, 2017). Although there is limited research into the school-based PA of children with CF, one recent study advises that teachers could incorporate PA into the daily routines of children with CF to improve their health outcomes (Valencia-Peris et al., 2021). Furthermore, research still suggests levels of PA during school hours for all children are not enough and schools should develop strategies to help children engage in more PA in the school setting (Grao-Cruces et al., 2020). This suggests that schools could offer PA opportunities for children with CF in an inclusive way by targeting all children. It is therefore important to gain parental perspectives on this, as they are the ones who manage the daily care routine for their children with CF and considering their experiences will be essential in what they consider safe, ethical and effective use of PA in the daily maintenance of their child’s CF. To the authors’ knowledge, this is the first study to investigate parental views of PA levels during the school day for children with CF. Therefore, the overall aim of this study is to explore parents’ perceptions of school-based PA for their children with CF.

## Materials and methods

### Participants

Parents of children with CF (*n* = 10) were purposely selected (Cohen et al., 2017), to take part in this study to explore perceptions of their children’s PA behaviours during the primary school day. The selection criteria were 1) having a child with CF, 2) their child is at primary school (4–11 years), and 3) they live in the UK. Participants were recruited via a social media platform and the recruitment strategy followed a recommended checklist for proposing social media recruitment (Gelinas et al., 2017). Parents were passively recruited through a closed parents of children with CF support group on social media by initially gaining permission from the administrators and then asking them to place a post in the group which explained the purpose of the research and the contact details of the researcher. The participants were from a range of regions in the UK including Wales, England, and Northern Ireland. Their children included a mixture of boys (*n* = 8) and girls (*n* = 2) with CF, with ages ranging from 5 to 10 years old (*M* = 8 years). All parents that agreed to take part were female. The study received ethical approval from Birmingham Newman University’s Research Ethics Committee (ref: S2019/019).

### Design

An IPA methodology was employed (Smith et al., 2022), that is based on the three concepts of phenomenology, hermeneutics and idiography. IPA is a version of phenomenology which accepts that it is not possible to gain direct access to a participant’s worldviews, but rather such an approach will always be affected by the researcher’s own views and interpretation of the participant’s experience (Willig, 2017). Hence, the researchers’ knowledge of children’s PA behaviours in a primary school environment (two of the research team are qualified primary school teachers) and one member of the research team has a child with CF, which will aid the methodological approach of IPA and overall interpretation of the participants’ lived experiences. Thus, an element of this research design could be considered as insider research, considering that the common definition of insider research is research that is undertaken within an organization, group or community where the researcher is also a member (Brannick & Coghlan, 2007; Hellawell, 2006; Hockey, 1993; Mercer, 2007). However, it has also been described as research that is undertaken by someone that has priori intimate or familiar knowledge of the group and may not be necessarily a member of that group (Hellawell, 2006; Merton, 1972). Insider research can be placed on a continuum (Fleming, 2018), this specific research project can be placed at the lighter end of the continuum in which the researchers will be interviewing strangers and have removed themselves as a member of the online support group. Measures were put in place to address any insider research bias following published guidelines (Fleming, 2018).

### Procedures

Stemming from this IPA methodology, the method of online semi-structured interviews (Smith et al., 2022) was conducted via Zoom in which the questions and topic were provided but the questions and sequence were adapted to the responses given by the interviewees; additionally, the researcher was able to ask follow-up questions or prompt the participants (Cohen et al., 2017). The interview guide was based on, but not exclusive to, the following sections 1). Understanding the main concepts under study (i.e., PA, CF), 2). Views on PA in children with CF, 3). Barriers and facilitators to PA in children with CF in the school environment, and 4). Views on training for schools in the promotion of PA in children with CF. As part of an IPA interview, the location should be left to the participant’s preference (Alase, 2017). However, as the interviews were conducted during the COVID-19 pandemic, the interviews took place on a video conferencing platform (Zoom), and although it cannot replace a face-to-face interview it does provide a viable alternative and produce some advantages in terms of opening up a wider participant pool (Archibald et al., 2019; Lo Iacono et al., 2016). Both researchers and participants have expressed general satisfaction with Zoom with evidence of its sustainability as a qualitative research data collection tool (Archibald et al., 2019) and using video “recordings” provides the additional benefit of not needing other equipment such as a Dictaphone (Lo Iacono et al., 2016). In addition, researchers have highlighted that Zoom’s ability to securely record video interviews is a key advantage for data management and protection (Archibald et al., 2019). All interviews lasted between 50 and 60 min. As interviews took place online, participants were asked to position themselves in a room free from distractions and use headphones to limit conversations being overheard. Consent was obtained via participants stating in an email that they provided fully informed consent and read and understood the participant information sheet. Participants were also asked to give verbal consent at the start of the interviews.

### Data analysis

Data were analysed using IPA (Smith et al., 2022), where a systematic analysis of each transcript took place. The first step involved reading and re-reading the transcripts; at this stage, initial notes were recorded. In IPA, it is advised that reading the transcript allows the researcher to begin a stage of active engagement with the data, with a focus on entering the participant’s lived experiences (Smith et al., 2022). In the second step, exploratory comments were produced, with a clear phenomenological focus. This then led to more interpretative hermeneutic insights being noted. The third step led to the development of experiential statements; here the focus was placed upon reducing the large amount of data to discrete phrases representing the large data set. This entailed breaking up the narrative flow of the interviews and fragmenting the hermeneutic cycle. The next stage of the analysis involved charting and mapping of experiential statements, looking for meaningful connections. The experiential statements were then clustered together to form personal experiential themes (PETs) (Smith et al., 2022). This systematic process was repeated for all individual interviews. The final stage involved looking for patterns across all PETs generated and creating a set of group PETs.

### Quality criteria checks

An IPA research study aims to collect quality data in order to produce transferable research findings (Alase, 2017). Therefore, the trustworthiness (Shenton, 2004) of the data was sought through the application of the following quality criteria checks: credibility (i.e., thick description of the phenomenon under study), transferability (detailed contextual information, stating boundaries of the study), and dependability (detailed description of the study).

## Results

All the themes outlined below in Table I are a representation of the Group Experiential Themes

**Table I.** Representation of group experiential themes.

First order themes	Importance of PA at school for children with CF	Barriers to school-based PA for children with CF	Facilitators of school-based PA for children with CF	Educate the educators on school-based PA for children with CF
Sub themes	− Time at school − PA part of daily routine − Parental pressures/treatment burden	− Teachers’ misconceptions − Emotional and -physical barriers − PA is not a priority	− Windows of opportunity − Seeing is believing − School promotes PA for all	− PA should be inclusive and personalized − More guidance needed − Health Care Plans

### Importance of physical activity for children with CF

‘Time at school, “PA part of daily routine” and “Parental pressures/treatment burden” all sit within the main theme of the 'Importance of physical activity for children with CF'.

### Time at school

Half of the participants outlined the amount of time their children spend at school and often that leaves little time outside of school hours for their children to be physically active. To them, the school environment is a key opportunity for their children to be physically active and further it is a need that they feel must be met. As expressed in the following quotes, “they don’t have time in the evening really to do a whole lot of exercise” (Participant 5), “children spend so much time at school, we can be really restricted, if you have a child with dyslexia or autism, they would get extra support, so a child with CF should get extra support at school” (Participant 8), “I sent my child to school with a Fitbit on and they were averaging about 600 steps a day, I was horrified” (Participant 3), “school takes up a massive part of when our children are not tired, you know in a day” (Participant 6).

### PA part of daily routine

The majority of participants discussed how being physically active should be an important part of their child’s daily routine to help keep them healthy. Some parents expressed the need for it to be integrated into the school day and other parents stated that it is something they take control of and deal with outside of school hours. For instance, “it’s just routine now, it’s just something that they do” (Participant 2), “keeping them healthy is important, I just don’t want my child to have to struggle in life and physical activity is going to keep them well” (Participant 10), “from literally the moment they could walk, I had them running, they were swimming from the age of 6 months just to keep their lungs clear” (Participant 7), and “it is something that we deal with outside of school, I don’t look at school as a place to manage their physical activity, however anything that he does do is a bonus” (Participant 7).

### Parental pressures/treatment burden

All participants described the pressures that having a child with CF can bring, including physiotherapy, nebulized medications and physical activity, diets and hospital admissions. As illustrated in the following participant quotes, “I’m rushing home for bedtime stuff and the physical activity side of things is limited to the weekend so we really do need schools to have PE, even if it’s just a couple of mornings a week” (Participant 6), “we wake up in the morning we do nebulizers, physio and exercise” (participant 1), “they are doing physio and medicines morning and night” (Participant 4), “they have five nebulizers, hypertonic saline twice a day, Colymicyn twice a day, Dnase once a day, four puffs on their inhaler before physio morning and evening, then they have two puffs of their purple steroid inhaler morning and night, for physio they do a combination of exercise, and they’ve got a vest and they do breathing treatments on their Acapella or Areobica”, It is incredibly difficult as a full-time working parent, I do solely rely on school, and their (child) swimming lessons on a Saturday morning” (Participant 6), “I would like the school to take more responsibility for it, I would like them to be more active in getting them active, so it takes the burden off me” (Participant 10).

### Barriers to school-based physical activity for children with CF

The three main barriers to engaging in school-based PA for children with CF were: “Teachers” misconceptions’, “PA not a priority” and “Children’s emotional and physical barriers”.

### Teachers’ misconceptions

Teachers’ misconceptions were a common thread throughout the interviews. Parents described how their children were treated as though they were vulnerable, which led teachers to encourage children to relax and take it easy rather than engaging in physical activity. As illustrated in the following parent quote:

I think sometimes teachers actually think that with lung conditions that they probably should be doing less exercise. I think the general misconception is you know that they’re vulnerable or they’re not very well. (Parent 9)

Parents described teachers as being nervous about having a child with a chronic lung condition in their care and expressed concern that teachers saw the CF rather than seeing the whole child and their capabilities. For instance, “It is important to not treat that child like they’re made of glass, I mean, they climbed Snowden at the age of five” (Parent 7) and “they kind of go to the CF rather than seeing the whole child” (Parent 6). One parent stated that their child was often kept in at lunchtime to complete work, which further restricted PA opportunities, i.e., “They keep him in over lunch time to do some of his work, which really annoys me because they would get much more benefit out of being in the playground” (Parent 1). Another parent highlighted teachers’ misconceptions around their child coughing whilst exercising, e.g., “The coughing thing, don’t be alarmed if they cough, it’s actually a good thing if they’re exercising, don’t think that you need to stop them unless they literally say they can’t do it anymore” (Parent 3).

### Emotional and physical barriers

Parents described how a range of emotional and physical effects from CF impacted upon their child’s well-being and was often a barrier for being active. For instance, one parent highlighted how their child was embarrassed by their body due to scars from operations which caused them to misbehave to avoid getting changed for PE in front of their peers, ‘my child’s finally told me that they are not comfortable with getting changed in front of other people in the classroom and that’s why they mess around because then they know they’ll get sent out and they won’t have to join in” (Parent 1). The same parent described how her child often gets teased at school for being “slower” than the other children, and they experience emotional upset due to frequently getting picked last for teams in PE and the playground.

They feel like they get a bit picked on, because they can’t keep up with everybody else, or if they’re not feeling their best, they will slow down a bit. So, they will not get, you know, picked for teams for football and stuff, they’ll be one of the last children to get picked because they’ll slow the rest of the team down. (Parent 1)

Further, a common finding was parents describing a range of physical effects of CF that could impact upon their children’s PA levels. Such as having a tight chest “if they genuinely feel their chest tightening or anything like that, then that’s a sign that there’s something lurking” (Parent 6), hydration “the biggie is always making sure that they keep hydrated obviously because they will dehydrate quicker than the other children” (Parent 2), tiredness “so depending on how well they are eating that can effective how tired they get” (Parent 4), and being cold (i.e., the cold air, or cold during swimming) “it’s breathing it in that affects them, on a cold morning”, “I actually bought them a wetsuit, because they used to get so like ridiculously cold when they used to have swimming lessons at school and outside of the school” (Parent 7).

### Physical activity is not a priority

From the interviews, it became apparent that parents’ felt that schools did not really see PA as a priority for children with CF. Parents expressed how physical education (PE) was not seen as an important subject and would often be cancelled or only have a small place in the weekly timetable. For example, “I mean obviously the schools in general, they have to have their two PE lessons, a week, which, in general, if you think of how much a child does in those isn’t actually very much” (Parent 7), “there’s a big push on maths and English, to the detriment of things like physical education but you know, they need physical education” (Parent 5), and “I do know for a fact that PE is the first thing that gets taken off the agenda whenever Christmas concerts come along or something, if the assembly hall is being used for something else” (Parent 5).

In relation to the parents’ priorities, although they viewed PA as extremely important, they would ensure that their children had other needs met in school in the first instance. For example, “the health care plan is all about Creon (digestive enzymes), making sure they have a high fat diet, making sure eating dinner and keeping away from mouldy things and you know stagnant water. It is my child’s responsibility that if they see a child with a cough or a cold they step away from them and those sorts of things but, no, no talk about physical activity” (Parent 6), “Are they going to get the food in, are they going to be near a child with a snotty nose, we’re more concerned about that, then we are about making sure that they’re doing their PE” (Parent 7), and “This year was mainly just getting the diet right, the Creon and getting that kind of cleanliness guidance down. I don’t think there’s anything specific about physical activity and as they go through the school it’s something that we will add in, I think, but there’s nothing specific at the minute” (Parent 2).

### Facilitators of school-based physical activity for children with CF

The main three facilitators of school-based PA for children with CF were: “Windows of opportunity”, “Seeing is believing”, and “School promotes PA for all children”.

### Windows of opportunity

A strong theme in the data, was the discussion of windows of opportunity during the school day for their children with CF. Some parents described how their children would start the day off with accumulating PA by active travel to school such as “they will run or cycle to school and back, I normally say run to that lamp post to sort of get them to run” (Participant 10), “so we walk to school, it’s only about 400 yards away” (Participant 3), and “we walk to school every morning and we are normally late but I don’t care” (Participant 5). Break and lunch times during the school day were another opportunity for children with CF to be physical active. For instance, “I think the majority of the exercise they get is from running around in the playground with their friends” (Participant 7), “they play cops and robbers in the playground, you know they do seem to run around” (Participant 5), “they will run around and play tag and stuff at breaktime and at dinner time it’s the same’ (Participant 1). Other windows of opportunity to be active during the school day for children with CF included PE lessons and after school clubs. For example, “In PE they do everything from football through to yoga and swimming” (Participant 6), “they have PE once a week for two hours, it’s an afternoon session and they come out sweating buckets, and I’m like, yeah you’ve had a great workout” (Participant 10), “they do PE twice a week and it involves a lot more running when they do it outside, they often say they get tired and out of breath” (Participant 3), and “when they do after school clubs like football or archery, they always say mommy you need to sign me up” (Participant 1).

### Seeing is believing

Parents expressed their belief in the value of being physically active in helping to manage their children’s CF symptoms. As highlighted in the following participant quotes, “we noticed that when they don’t exercise their lung function drops” (Participant 8), “I can tell if they haven’t done proper exercise for days as they get like a throat cough” (Participant 7), “They are definitely healthier and better when they exercise” (Participant 3), “they can wake up in the morning and have a really wet cough, but then we will do nebulizers, physiotherapy and run around and by the time they have done all of that they sound quiet dry” (Participant 1), and “my child’s teacher has a better understanding of the benefits of being active, so they did push my child towards doing sports, the teacher has really gone out of their way to learn as much as they can” (Participant 9).

### School promotes PA for all children

Three participants described how understanding and promoting physical activity for all children would help to provide PA opportunities for their children with CF. For example, “there is obesity and everything, so all kids will thrive from physical activity, every kid will benefit from it … even if my child didn’t have CF I still feel I would say being active is important” (Participant 5), “I mean they are quiet good at school, they do push physical well-being, they follow the children’s university thing, so they can log their hours of physical activity, they are quiet good for it” (Participant 6). One parent discussed the benefit of PA for all children’s mental health, well-being and academic engagement, “if they did whole school based physical activities prior to learning, the comfort and peace that offers to somebody before they sit down and engage with learning is you know, you can’t put a price on that” (Participant 8).

### Educate the educators

Under the main theme of Educate the Educators are the following three sub themes: “Inclusive and personalized”, “More guidance needed”, and “Individual Health Care Plans”.

### Inclusive and personalised

All participants expressed the importance of PA during the school day for their children with CF, but that this should be achieved in an inclusive way. Parents felt that they did not want their children to be made to feel different or singled out in anyway. Some parents expressed how their children would hide their CF from friends. They wanted the school to provide PA opportunities but for all children, rather than just focusing on their child. For example, “they didn’t want to feel different from her friends, they didn’t want people to know that they have CF, so it would have to be some sort of whole group activity rather than her doing it on her own” (Participant 8), “I’m keen for my son to think that doing physical activity is normal for him and normal for everyone” (Participant 6), “It is very much an invisible disease, so it’s more a case of him doing what his friends do” (Participant 7), “but why should they be treated any different” (Participant 9), “I would want her to be treated the same as everyone else, she is quiet funny about being singled out” (Participant 3), “I think if it was done in a way that no-one else really noticed then that would be the best way” (Participant 4), “I don’t want it to be like, my daughter has to carry on running and all the other children can sit down, I just don’t want her to feel like she is singled out for anything” (Participant 10). Parents also expressed that every child’s condition and parental views with CF can be different, therefore a personal approach and consultation was needed in each case. As described in the following quote “I think it is an individual case per child because for my son he is very fit and well and never gets out of breath but for some children with CF they can struggle when exercising and may not want to cough up lots of stuff in front of other children” (Participant 7), “you know, blanket guidance doesn’t really work for something with CF because it is quiet an individual condition at the heart of it” (Participant 2), and “it’s the clinical team that should have a physical activity programme for that child because every child is different” (Participant 3).

### More guidance needed

Although participants wanted an individual approach to PA for their children with CF, they also felt like more guidance was needed for schools in terms of the promotion of PA for children with CF, e.g., “there isn’t any guidance that I’ve come across, I know they say that exercise is important, I think there should be some sort of guidelines to help the schools and parents” (Participant 6), “schools possibly need more guidance, I know my CF team hold an open day and do a presentation for schools” (Participant 3), “It would be great if there was support to give to the school in the promotion of physical activity, especially for children with chronic lung conditions” (Participant 5), “I think if schools promoted it that would be helpful, instead of saying it’s fine for the child with CF to join in, it could actually be more like this is really beneficial for the child with CF” (Participant 4). Another parent outlined the need to “educate the educators” (parent 6) as teachers and schools were often in the dark with limited knowledge of CF.

### Individual health care plans

Participants described how their child had an individual health care plan (IHCP) for managing their CF at school. Participant 8, stated that the IHCP was “wishy washy” and not that helpful, “they could be much more helpful for children in schools” (Participant 8). One parent described how they wouldn’t want PA written into their child’s IHCP, “It’s not something that I would want anyway, actually, you know, but if it was written into the health care plan, then it would be to ensure that all children were taking part in PA opportunities” (participant 8). Another parent described the sense of being overwhelmed by the number of things they have to remember to keep their child well, that sometimes PA can get a little lost, “actually the important message of exercise, it gets lost because you are told so many things from the physios” (Participant 6).

## Discussion

The purpose of this study was to explore parents’ perceptions of PA behaviours of their children with CF during the primary school day. The results of this study will be discussed according to the four group experiential themes, identified from the data analysis and in the context of previous studies, theory, and government documents. Finally, a set of recommended guidelines will be provided.

### The importance of school-based physical activity for children with CF

All parents expressed the importance of PA in their children’s daily routine and although there can be various tolerances in relation to children with CF and the amount of PA they engage in, studies evidence the importance of an active lifestyle for children with CF (Puppo et al., 2020; Williams et al., 2010). Taking into consideration children spend a large portion of their day at school, it is not surprising that some parents felt the school should take some responsibility for ensuring their children were engaged in PA during the school day. Parents described in detail the daily burden of CF in relation to medications and treatments, in particular physio and airway clearance. This finding aligns with large-scale survey data, which reported the treatment burden for people living with CF is substantial and multifactorial, with the median total daily time for treatments being two hours (Davies et al., 2020). There is often little time outside of the school day for treatments, with homework often placing extra burdens on families of children with CF. Therefore, parents expressed that their child with CF should receive support from the school in terms of their health condition and daily treatments, just as other children do with additional needs that relates to their education. Current educational policies advocate for inclusivity and equal opportunities in learning and health practices within schools (Department for Education, 2014b). However, our study suggests that the structure, organization and pedagogy in schools, can restrict daily PA opportunities for children with CF. This oversight can lead to indirect discrimination under the Equality Act 2010 (Department for Education, 2014a), given failing to accommodate the unique needs of children with CF can restrict their access to the health needs of PA. Schools should, therefore, consider bespoke PA integration strategies that are cognizant of the medical and social needs of children with CF, ensuring that these strategies are embedded within individual Health Care Plans.

### Barriers to school-based physical activity for children with CF

Emotional and physical barriers to school-based physical activity for children with CF were described by parents. They stated how sometimes their children would feel embarrassed about visual body scars and would avoid doing PE, so they would not have to get changed in front of their peers. Further, parents described that their children would get teased for being slow which would result in withdrawal from PE lessons and any physical activities during break times. A systematic meta review of experiences of children with chronic illnesses at school (Lum et al., 2017), reported that several studies described children with chronic illnesses (including cystic fibrosis) often being teased and bullied in relation to their body image and physical functioning. The review also reported greater disease severity being linked to poorer social relationships with peers in the school setting (Lum et al., 2017). In addition, parents described teachers’ common misconceptions regarding their children’s physical ability due to having a chronic lung condition. Concepts like this have been previously reported such as the “protected child syndrome” (Thornton, 1997) and the “vulnerable child syndrome” (Schmitz, 2019), which describe a phenomenon in which children are perceived as being at a higher risk than is warranted. These perceived risks by teachers could contribute to children’s disease severity, as if children are being restricted from engaging in physical activity this will be a potential determinant to a child’s health. However, teachers frequently report inadequate training in how to support children’s health and psychosocial needs (Daly et al., 2022). Appropriate training can help to address teachers’ misconceptions about children with CF and provide them with the knowledge to support all children with positive social relationships, which should in turn help children with CF to engage in daily PA without the fear of being teased or bullied.

Furthermore, perceived physiological barriers to children with CF engaging in school-based physical activity were evident, for instance, parents described how if their children were poorly, then this could impact upon their ability to take part in physical activity. A recent systematic review (Denford et al., 2020) also found fluctuating health as a common barrier to PA, with the systemic review describing the limiting impact of being ill with CF, with people finding it difficult to complete daily routine tasks such as brushing their teeth. Therefore, schools need to be mindful that the amount of daily PA children with CF can engage in, may change depending on a child’s current health status and their fitness levels (West et al., 2019). Other discussed physiological barriers to children with CF participating in PA included the need to stay hydrated, becoming easily cold, and frequent coughing. There is currently no known research that reports children with CF being affected by cold temperatures more than other children without CF. However, as children with CF are more likely to be underweight, this may increase their likelihood of being more susceptible to the cold weather and colder school-based activities such as swimming, especially if limited PA is taking place.

### Facilitators for school-based physical activity

Parents described key windows of opportunity during the school day in which they believed that their children with CF took part in physical activity, for instance active travel to school, break times, physical education and extra-curricular sports clubs. However, despite these daily opportunities to take part in PA, a recent study evidenced that both children with CF and children without CF do not meet their daily physical activity guidelines (Valencia-Peris et al., 2021). Further gender analysis in the study revealed that girls with CF were the least active group, with only 16.7% meeting the PA guidelines. The study also indicated compensatory effects between sedentary behaviour and physical activity, especially in girls with CF. Therefore, it has been recommended that teachers and caregivers promote a reduction in sedentary behaviour for children with CF (especially girls) to improve overall health outcomes. Parents also described how they noticed their children’s health outcomes improve if they took part in regular PA and exercise, with some parents stating how their child’s teacher has a good understanding of the benefits of physical activity for their child with CF. These observed health outcomes related to PA engagement, are supported by previous research (Puppo et al., 2020), with PA being described as an important part of CF care leading to better health-outcomes. Research often indicates that if teachers see a positive change in student outcomes, because of changes to their practice, they are more likely to sustain their new practice, i.e., encourage children with CF to take part in PA during the school day (Kern et al., 2019).

A discussed facilitator of PA for children with CF is when school’s promote daily PA opportunities for all children. Parents expressed the importance of PA for all children and the related health benefits including physical, mental, social and overall well-being. The benefits of engaging in PA for all children have been well documented (Chaput et al., 2020; Janssen & LeBlanc, 2010), including cardiorespiratory fitness, muscle fitness, bone health, and cardiometabolic health. The research also evidences that PA has positive effects on cognitive function, academic outcomes and reduces depressive symptoms (Chaput et al., 2020). If the school setting is able to provide daily PA opportunities for all children, then this would not only benefit every child but provide PA opportunities for children with CF in an inclusive way. As advocated by recent literature, inclusion for children with additional needs can often be an illusion (Webster, 2022). Children with CF should not be singled out. Schools should be ensuring that the PA opportunities they provide for children with CF are closely aligned to the PA experiences they provide for children who do not have CF. As PA benefits all children.

### Guidance for school-based physical activity for children with CF

Parents expressed more guidance was needed to support children with CF engaging in daily PA at school. Some parents referred to the UK’s Cystic Fibrosis Trusts’ pre-school and primary school information pack’ (Cystic Fibrosis Trust, 2016) but felt additional information could be provided on PA. Parents also expressed that the importance of their child engaging in PA could often get lost amongst all the other things they wanted the school to instil such as diet, medication, and infection control. They also voiced the need for teachers and schools to be educated on the importance of PA and how they could support their children to be more active in the school setting. The UK’s DfE’s statutory guidance on “Supporting Children in Schools with Medical Conditions”, states that schools should ensure children are properly supported at school, including having full access to physical education. Further outlining that school leaders should consult health professionals, parents, and children to fully understand how children can be effectively supported at school (Department for Education, 2014b). Even though parents wanted their child to be involved in more physical activity at school, they strongly believed that this should be in an inclusive way as they did not want their child to be singled out and made to feel different. The need for a personalized approach was also evident as parents felt blanket guidance would not be appropriate. This is supported by the UK’s DfE’s statutory guidance which states that children with the same condition may require very different support (Department for Education, 2014b). Although a personalized approach is needed for PA recommendations and children with CF, research does evidence that PA is an important part of care for all children with CF (Puppo et al., 2020). Further, the UK CF Trust recommends that PA should be encouraged in the school environment. Parents believed that in order for this personalized approach, teachers needed to be educated on the benefits of PA for children with CF, along with guidelines that could be adapted to suit the individual needs of each child.

Although parents did report that their children had individual Health Care Plans, they were often vague and did not outline anything in relation to PA guidance. This links with the work of Nel Noddings on the ethics of care (Noddings, 1986). Schools should not be quick to complete Health Care Plans without considering the expressed needs of parents and children. For children with CF, when they go to school, they are dependent on the care of teachers. If we want children with CF to thrive and grow, we must care for them in the school setting. They must be given opportunities to engage in daily PA. With the combined limited knowledge of CF and vague Health Care Plans, it is not surprising that some parents commented that teachers viewed their children as being fragile, which can be linked to the concept of the “protected child syndrome” (Thornton, 1997). These findings reinforce the need to bridge the gap between policy and practice, as schools need actionable guidelines that are both feasible and tailored to the needs for children with CF.

Considering the findings from this study on parents’ perceptions of primary school-based PA for their children with CF, prior research, UK government documents, and the UK’s CF Trust information, the following guidelines are recommended:

1. School leaders should consult with health care professionals, parents, and children, when considering the type, duration, and intensity of PA that children with CF should engage in during the school day.
2. Advice regarding school-based PA for a child with CF should be included in their individual Health Care Plan, and this plan should be reviewed at least annually. All school staff teaching and/or coaching children with CF during PE, PA and/or school sport should be fully aware of and implement the content of the individual Health Care Plan.
3. Children with CF should be afforded opportunities for school-based PA in an inclusive way. PA is beneficial for all children, not just children with CF. Consider key windows of opportunity during the school day for all children, e.g., active transport to school, breaktimes, PE, extra-curricular clubs, and active lessons.
4. PE lessons and extra-curricular activities should be active (50–80% moderate to vigorous PA), safe, and enjoyable for children with CF, this will help to engage them now and to establish a positive, long-life relationship with PA.
5. Reduce periods of sedentary behaviour during the school day, e.g., sitting in class.
6. On PE days, allow all children to come to school in their sports kit. Children with CF may feel body conscious, especially in relation to their weight (both under and overweight), have body scars and/or ports. This will also help to maximize the time spent in PE.
7. When taking part in PA, PE and school sport, children with CF may need to cough, this is a good thing, as they are clearing mucus from their lungs. Have tissues or a cup ready in case they need to clear out mucus.
8. Ensure children with CF stay hydrated before, during and after taking part in physical activity, especially on warmer days.
9. Children with CF may get cold quickly, ensure they have extra clothing if needed and consider thermal swimsuits for school swimming lessons. On cooler outdoor PE days and during swimming lessons, ensure children with CF are active and not stood around getting cold.
10. If children with CF become unwell, this can impact upon the amount of PA they can engage in, consult with parents and look for signs of tiring and needing rest.

## Limitations and future research directions

The strengths of this study lie in the rich data set of parents of children with CF from across the UK (including, England, Wales and Northern Ireland), and the ages of their children ranged from 5 to 10 years. Furthermore, these findings support transferability of the results across the UK. However, a limitation of this study is that, only mothers volunteered to take part, therefore fathers’ experiences have not been considered. Future directions for research would be to collect the experiences and perceptions of CF healthcare providers, teachers and children with CF related to school-based PA opportunities in maintaining health and reducing the burden of care on families.

## Conclusion

Overall, the findings of this study evidence that although parents recognized the benefits of school-based PA for their children with CF, systemic barriers in school settings often inhibit its implementation. This aligns with recent health policies aimed at increasing activity levels among children but highlights a gap for specific provisions for children with CF. The integration of PA into the school setting should be seen as a component of CF care that extends beyond the medical setting and the home environment. This would promote a broader understanding of inclusivity in the school environment, showing that a well-rounded education includes physical health maintenance, particularly for children with CF. Therefore, guidelines have been developed, to inform parents, primary schools and CF healthcare professionals to help children with CF engage in inclusive daily PA, with the hope of maintaining health-outcomes for children with CF and reducing the burden of care on families.

## Data Availability

The data presented in this study is restricted due to the sensitive nature of the interview content and ethical considerations. However, whilst unable to share the data, the authors are able to address any inquiries regarding the data set and content.
